# Peeling the onion: the outer layers of *Cryptococcus neoformans*

**DOI:** 10.1590/0074-02760180040

**Published:** 2018-05-07

**Authors:** Daniel P Agustinho, Liza C Miller, Lucy X Li, Tamara L Doering

**Affiliations:** Washington University School of Medicine, Department of Molecular Microbiology, St. Louis, Missouri, USA

**Keywords:** Cryptococcus neoformans, polysaccharide capsule, cell wall, plasma membrane

## Abstract

*Cryptococcus neoformans* is an opportunistic fungal pathogen that is ubiquitous in the environment. It causes a deadly meningitis that is responsible for over 180,000 deaths worldwide each year, including 15% of all AIDS-related deaths. The high mortality rates for this infection, even with treatment, suggest a need for improved therapy. Unique characteristics of *C. neoformans* may suggest directions for drug discovery. These include features of three structures that surround the cell: the plasma membrane, the cell wall around it, and the outermost polysaccharide capsule. We review current knowledge of the fundamental biology of these fascinating structures and highlight open questions in the field, with the goal of stimulating further investigation that will advance basic knowledge and human health.

*Cryptococcus neoformans* is an opportunistic fungal pathogen that causes severe infection of the central nervous system. Inhalation of this microbe, as either a spore or desiccated yeast cell (Giles et al. 2009), causes a pulmonary infection that in immunocompetent individuals is minimally symptomatic, although it may remain latent for extended periods of time (Kwon-Chung et al. 2014, Ballou and Johnston 2017). In severely immunocompromised individuals, however, *C. neoformans* can disseminate from the lungs and cross the blood-brain barrier (Santiago-Tirado et al. 2017), causing an often-lethal meningoencephalitis. Close to 220,000 cases of cryptococcal meningitis are reported annually. These result in over 180,000 deaths worldwide, including 15% of all AIDS-related deaths (Rajasingham et al. 2017). Mortality rates range from 10 to 75% (Day et al. 2013, Jarvis et al. 2014), even with carefully developed treatment regimens (Perfect et al. 2010), due to challenges that include drug toxicity, efficacy, cost, and availability. Increasing drug resistance has also been reported (Sionov et al. 2013, Chen et al. 2015, Smith et al. 2015). Clearly, improved therapies are needed to combat this infection.

The outer layers of *C. neoformans* have unique features that may offer directions for drug discovery. These consist of three concentric structures: the capsule, cell wall, and plasma membrane (Fig. 1A, left). The outermost layer, the polysaccharide capsule, is the hallmark of this organism and is required for virulence. This highly dynamic structure modulates fungal interactions with immune cells. Capsule components are also shed into the environment, where they further influence the host response and may be exploited for diagnosis and monitoring of cryptococcal infection (Alspaugh 2015). Both capsule thickness and shedding are tightly regulated in response to environmental conditions (Kumar et al. 2011, Maier et al. 2015).

**Figure:**
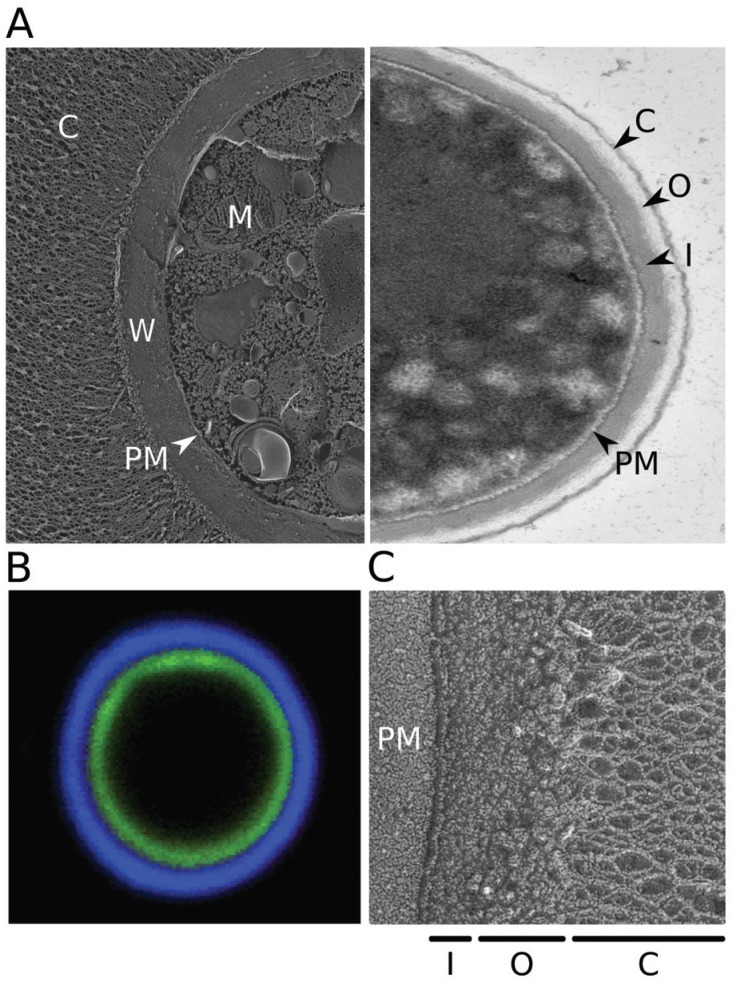
(A) Electron micrographs of *Cryptococcus neoformans.* Left, quick-freeze deep-etch image of cells grown in capsule-inducing conditions (as in Haynes et al. 2011); right, transmission image of cells grown in rich medium (YPD), which yields only thin capsules. C: capsule; W: cell wall; PM: plasma membrane; M: mitochondrion; O: outer cell wall layer; I: inner cell wall layer. (B) Fluorescent micrograph highlighting the cell wall and capsule. Cryptococcal cells were induced to form capsule and stained with florescein (green) to label the cell wall and monoclonal antibody 2H1 (blue, generously provided by Arturo Casadevall) to label the capsule, as in Pierini and Doering (2001). (C) Quick-freeze deep-etch image highlighting the two layers of the cell wall.

Below the capsule, and anchoring it, lies the fungal cell wall. This complex structure surrounds the plasma membrane and helps the cell withstand environmental challenges such as osmotic and mechanical stress. It is composed of glucans, chitin, chitosan, and glycosylated proteins (Gow et al. 2017). Melanin pigment associated with the cell wall further helps *C. neoformans* resist environmental stress and antifungal drug toxicity.

The innermost of the three layers we will consider is the plasma membrane. Unique features of fungal membranes are already the targets of several important classes of antifungal drugs, the polyenes and azoles. The plasma membrane is also the site of multiple proteins implicated in fungal virulence and has been implicated in the production of extracellular vesicles with potential roles in virulence (Rodrigues et al. 2014, Brown et al. 2015, Joffe et al. 2016, Rella et al. 2016).

The unique features of the cryptococcal capsule, cell wall, and plasma membrane demonstrate fascinating fundamental biology that may potentially be exploited for therapy. Below we review current knowledge of these structures and their synthesis, and highlight some open questions in the field. We do not address the regulation of these structures in this short article, but refer interested readers to recent reviews that address this important topic (Doering 2009, Gilbert et al. 2011, O’Meara and Alspaugh 2012, Bahn and Jung 2013, Kwon-Chung et al. 2014, Srikanta et al. 2014, Alspaugh 2015, Rella et al. 2016, Gow et al. 2017).

*The capsule* - The polysaccharide capsule is a major cryptococcal virulence factor, which impedes the host immune response and is required for fungal survival within the host. This structure is composed of two polymers, glucuronoxylomannan (GXM) and glucuronoxylomannogalactan (GXMGal), along with trace mannoproteins (Cherniak et al. 1998, Doering 2009). GXM, which typically has a molecular weight in the millions, consists of an a-1,3-linked mannose backbone substituted with glucuronic acid and xylose (Cherniak et al. 1988, Cherniak et al. 1998, Heiss et al. 2009). GXMGal, roughly an order of magnitude smaller, is a galactan with galactomannan side chains bearing a variable number of xylose residues; the galactose backbone also bears galactofuranose (all sugars are pyranose where not specified) (Heiss et al. 2013, Previato et al. 2017). Mannose residues of both polymers may also be O-acetylated (Gates-Hollingsworth and Kozel 2009, Previato et al. 2017). Based on analysis of shed capsule, GXM constitutes ~90% of the capsule mass and GXMGal the remainder (Cherniak and Sundstrom 1994), although this may differ for surface-associated capsule.

The reactions that incorporate individual monosaccharides into complex polysaccharides like those of the capsule or cell wall use activated sugar molecules, usually in the form of nucleotide sugars, as donors. These compounds are mainly synthesized in the cytosol, although most glycan biosynthesis, including the formation of capsule polysaccharides (Yoneda and Doering 2006), occurs in the lumen of the secretory pathway. For this to occur, the precursors must be moved into the synthetic compartment by specific nucleotide sugar transporters (NSTs) (Caffaro and Hirschberg 2006, Hadley et al. 2014). *C. neoformans* transporters have been identified for all of the predicted capsule precursors: GDP-mannose (Cottrell et al. 2007, Wang et al. 2014), UDP-galactose (Cottrell et al. 2007, Moyrand et al. 2007, Wang et al. 2014, Li et al. 2017), UDP-galactofuranose (Li et al. 2018a), UDP-glucuronic acid (Li et al. 2018b), and UDP-xylose (Li et al. 2018a).

Once nucleotide sugars are transported into the lumen of secretory organelles, they serve as substrates for specific glycosyltransferases, which mediate the synthetic reactions that form capsule polysaccharides. The complex structures of GXM and GXMGal suggest the involvement of multiple such enzymes. However, only a single glycosyltransferase, Cxt1, has been directly implicated in capsule synthesis. This β-1,2-xylosyltransferase is required to produce fully xylosylated GXM and GXMGal (Klutts et al. 2007, Klutts and Doering 2008). Two other cryptococcal glycosyltransferases have been biochemically characterized (Sommer et al. 2003, Reilly et al. 2009, Reilly et al. 2011), but only one of them has specificity appropriate for a role in capsule production, and deletion of the corresponding gene does not alter capsule composition (Sommer et al. 2003). It remains to be determined whether this reflects the presence of compensating activities or indicates that this enzyme does not participate in capsule synthesis. The many other activities required for capsule synthesis (Bose et al. 2003, Klutts et al. 2006, Doering 2009) are likely encoded by some of the ~70 putative glycosyltransferase genes observed in the *C. neoformans* genome (Cantarel et al. 2009, Lombard et al. 2014); future work will be required to identify them.

Most glycan synthetic machinery is localized to the Golgi, where elaboration of core glycans on lipids and proteins occurs and capsule is synthesized (Yoneda and Doering 2006); Cxt1 also resides in this compartment (Klutts et al., unpublished observations). From here, the classical secretory pathway transports capsule material to the cell surface (Yoneda and Doering 2006). There are several models for the subsequent incorporation of newly synthesized polysaccharides into the existing capsule, which propose that this material is incorporated either at its inner face, near the cell wall (Pierini and Doering 2001, Cordero et al. 2013), or at the outer edge of the structure (Zaragoza et al. 2006).

Although capsule polysaccharides are generally described as linear polymers, they may also contain branches, as suggested by their viscosity and shape factor (Cordero et al. 2011). Increased branching has been reported to confer increased resistance to oxidative stress, inhibit nitric oxide production by macrophages, and enhance fungal survival in serum (Cordero et al. 2011). Interestingly, *Cryptococcus liquefaciens,* which has a capsule that is chemically identical to that of *C. neoformans* although with no evidence of branching, is not as adept at resisting predation by amoeba, a potential environmental host of Cryptococci (Araújo et al. 2012). However, this difference in intracellular survival does not hold in mammalian macrophages, suggesting that the biological activity of capsular polysaccharide may be context-dependent (Araújo et al. 2017) or other factors may be involved. The details of branch formation and its role in host-pathogen interactions are still unclear, but promise to be exciting areas of study.

Mannoproteins comprise a small fraction of the capsule mass (Cherniak and Sundstrom 1994). These polypeptides appear mainly in the inner region of the capsule, close to the cell wall (Jesus et al. 2010); they may represent an integral part of the capsule or perhaps secreted proteins that are in transit through it. These predominantly cell wall proteins are discussed further below.

*The cell wall* - Moving inward from the capsule, the next protective barrier that surrounds *C. neoformans* is the cell wall (Fig. 1B). This structure is essential for cell viability, because it protects the fungus from osmotic and other environmental stresses; it also serves as a scaffold for the capsule. The cell wall is composed of a- and β-linked glucans (glucose polymers), chitin (a polymer of β-1,4-N-acetyl-glucosamine), chitosan (deacetylated chitin), glycoproteins, and, in the presence of appropriate precursors, the pigment melanin (Agustinho and Nosanchuk 2017). Because the wall contains multiple components that are not shared with mammalian hosts, it has been the focus of much research for the development of antifungal compounds.

The cryptococcal cell wall is arranged in two layers. In quick-freeze deep-etch electron micrographs the inner layer appears more striated and the outer one more particulate (Fig. 1C); they also differ in electron density on thin section electron micrographs (Fig. 1A, right). The inner layer is composed of β-glucans and chitin; mannoproteins and melanin are also most abundant in this layer although they occur throughout the cell wall (Vartivarian et al. 1989, Wang et al. 1995). The outer layer mainly contains a- and β-glucans (Reese et al. 2007).

Each of the wall components has a specific role in maintaining cell wall structure and function. β-1,3-glucan underlies the cell wall framework. It occurs as long polymers (James et al. 1990), with short β-1,6-glucan branches that crosslink the polymers to each other, as well as to chitin and glycoproteins (Reilly and Doering 2009, Gilbert et al. 2011, Gow et al. 2017). Chitin and chitosan contribute to maintaining the integrity and flexibility of the wall structure (Banks et al. 2005), while chitin and chitooligomers have been implicated in capsule architecture (Banks et al. 2005, Rodrigues et al. 2008, Fonseca et al. 2009). a-1,3- glucans are required for tethering the polysaccharide capsule to the cell (Reese and Doering 2003, Reese et al. 2007).

Unlike capsule components, cell wall polysaccharides are made at the plasma membrane and extruded through it. Once outside the cell, they associate with each other and with cell wall proteins that have exited via the secretory pathway. Some of these interactions are directed by branching or cross-linking enzymes, which together establish the complex wall structure (Gilbert et al. 2011, Free 2013, Gow et al. 2017).

β-1,3-glucan is synthesized at the plasma membrane from UDP-glucose by Fks1. The antifungal drug caspofungin (Kartsonis et al. 2003) targets Fks1 in other yeasts, but notably is not effective against *C. neoformans*, even though it inhibits the cryptococcal enzyme *in vitro* (Maligie and Selitrennikoff 2005). The synthases Skn1 and Kre6 participate in formation of β-1,6-glucan, although their specific biochemical roles are not known; deletion of the corresponding genes also perturbs capsular architecture, likely due to disorganization of the underlying cell wall (Gilbert et al. 2010). Finally, a membrane-associated alpha glucan synthase [Ags1; (Reese and Doering 2003)] forms a-1-3-glucan.

Chitin is made at the plasma membrane by a family of chitin synthases. Although no individual family member is essential for *C. neoformans* viability, the deletion of genes encoding chitin synthase 3 (Chs3) and a chitin synthase regulator (Csr2) drastically impairs cell wall integrity (Banks et al. 2005). Unlike in other fungi, most of the chitin in *C. neoformans* is deacetylated to form chitosan, a polymer that confers extra flexibility on the cell wall. Cells from strains lacking all three chitin deacetylases (Cda1, Cda2, and Cda3) have no cell wall chitosan and exhibit defects in cell integrity (Baker et al. 2007). Chitosan is also essential for virulence (Baker et al. 2011), a feature that has been successfully exploited in using a chitosan-deficient strain of *C. neoformans* to induce robust protective immunity in a murine model of infection (Upadhya et al. 2016).

Another component of the cell wall that has been implicated in virulence is melanin, a negatively-charged polymeric and hydrophobic pigment made from phenolic or indolic precursors (Nosanchuk and Casadevall 2006). This material is associated with the cryptococcal cell wall in a chitin-dependent manner (Baker et al. 2007, Camacho et al. 2017). Melanization enhances cryptococcal survival within natural predators, such as amoebae (Steenbergen et al. 2001) or nematodes (Mylonakis et al. 2002). Disruption of its synthesis during infection reduces cryptococcal dissemination (Noverr et al. 2004) and virulence (Salas et al. 1996), possibly due to melanin-mediated inhibition of phagocytosis and modulation of host cell cytokine responses (Huffnagle et al. 1995, Mednick et al. 2005). Melanization also increases resistance to antifungal compounds such as amphotericin B and caspofungin (Wang and Casadevall 1994a, Martinez and Casadevall 2006), and to environmental stresses, including host oxidative and nitrosative responses (Wang and Casadevall 1994b).

Many proteins are present in the cell wall, most of them heavily modified with N- and O-linked glycans (Klutts et al. 2006, Levitz and Specht 2006, Doering 2009, Reilly et al. 2011). The majority of these originate as plasma membrane localized glycosylphosphatidylinositol (GPI)-linked polypeptides, which are transferred, along with part of their anchor glycan, to covalent linkage with cell wall β-1,6-glycans (Orlean and Menon 2007, Muniz and Zurzolo 2014). Computational analysis predicts over 50 GPI-linked proteins encoded by the cryptococcal genome (de Groot et al. 2005, Loftus et al. 2005, Levitz and Specht 2006); some of these have been confirmed in studies of the *C. neoformans* secreted proteome (Eigenheer et al. 2007). Other proteins associate with the cell wall via non-covalent interactions, linkage to β-1,3-glucan (Yin et al. 2008, Karkowska-Kuleta and Kozik 2015, Gow et al. 2017), or disulfide bonds to polypeptides that are themselves covalently bound to structural glycans (de Nobel and Lipke 1994, Jaafar et al. 2003); these processes are less well studied.

Multiple cell wall proteins have key functions in cryp-tococcal biology. Some have been implicated in the dynamic responses of the wall to environmental conditions, such as the GPI-linked β-glucanase Gas1 that acts in β-1,3-glucan remodeling (Levitz and Specht 2006, Eigenheer et al. 2007). Another important GPI-linked protein is phospholipase B1 (Plb1), which has been implicated in *C. neoformans* virulence (Siafakas et al. 2007, Maruvada et al. 2012). Cell wall mannoproteins, small amounts of which can be found in the capsule (see above), are often 80-90% mannose by mass (Mansour and Levitz 2003). They are highly immunogenic (Levitz and Specht 2006, Wozniak and Levitz 2009) due to their activation of the mannose receptor on dendritic cells and consequent activation of T-cells, which leads to a protective immune response against *C. neoformans* (Specht et al. 2007, Dan et al. 2008a, Dan et al. 2008b). These proteins are being explored as an adjuvant component of a vaccine for cryptococcosis (Chow and Casadevall 2011, Levitz et al. 2015).

Several cryptococcal mannoproteins have been studied in depth. The first one described, MP98, was shown to be involved in T-cell activation (Levitz et al. 2001) and turns out to be the same protein as the chitin deacetylase Cda2 discussed above (Gilbert et al. 2012). This protein, despite originating as a GPI-anchored membrane protein, associates with the cell wall in a manner that is independent of its GPI structure and β-1,6-glucans; the enzymatic activity is associated with the membrane form (Gilbert et al. 2012). Other mannoproteins that have been studied include MP88, involved in T-cell activation (Huang et al. 2002), and others (MP84 and MP115) that have homology to chitin deacetylase and carboxylesterase proteins (Biondo et al. 2005). MP84 has been reported to mediate adhesion of *C. neoformans* yeasts to lung epithelial cells, suggesting a role early in infection (Teixeira et al. 2014). Finally, the mannoprotein Cig1 participates in iron uptake and contributes to virulence in a mouse model (Jung et al. 2006, Cadieux et al. 2013). Future studies of these and other cell wall proteins will further illuminate the synthesis and function of this complex structure, and may also advance efforts to develop vaccines or therapies.

*Plasma membrane* - Beneath the polysaccharide capsule and cell wall is the plasma membrane (Fig. 1), which serves as a barrier to the passage of hydrophilic molecules (van der Rest et al. 1995). Fungal membranes are composed of sterols, glycerophospholipids, and sphingolipids (Ejsing et al. 2009, Singh et al. 2017), although these differ in many respects from their mammalian counterparts. The plasma membrane also contains proteins, which maintain their association with the membrane via transmembrane domains, GPI anchors, or various lipid modifications (Santiago-Tirado and Doering 2016). Membrane structure and composition are dynamic, and vary with the fungal species analyzed and the environmental conditions (Singh and Prasad 2011, Xia et al. 2011).

Fungal membranes differ from those of mammals in containing ergosterol in place of cholesterol. This feature has been exploited by two major classes of antifungal drugs, Amphotericin B, which binds ergosterol, and the azoles, which inhibit its synthesis. *C. neoformans* also produces glycosylated ergosterols, termed sterylglycosides (SGs) (Weete et al. 2010, Rella et al. 2016). Although the enzyme(s) responsible for SG synthesis has not been identified (Warnecke et al. 1999), a glucosidase involved in SG degradation (termed EGCrP2 or Sgl1) is known. Deletion of the corresponding gene yields growth arrest, abnormal budding, and abnormal vacuole morphology (Watanabe et al. 2015); it also eliminates virulence in a murine model (Rella et al. 2015). Interestingly, inoculation with this mutant protected mice against subsequent lethal doses of *C. neoformans* H99 and *Cryptococcus gattii* R265 (Rella et al. 2015).

Yeast glycerophospholipids resemble those of higher eukaryotes, although their fatty acid composition may vary, while glycosphingolipids are more distinct. One of the latter that has been studied in detail in *C. neoformans* is glucosylceramide (GlcCer) (Nimrichter and Rodrigues 2011), which influences cryptococcal pathogenicity and is required for normal growth, resistance to alkaline conditions, spore production, and germination (Del Poeta et al. 2014). GlcCer is formed by the enzyme glucosyl-ceramide synthase 1, which transfers glucose from the nucleotide sugar UDP-glucose to a ceramide backbone (Rittershaus et al. 2006, Rella et al. 2016); disruption of the corresponding gene abrogates growth and virulence (Rittershaus et al. 2006). Notably, fungal GlcCer differs from that of other eukaryotes by the presence of a methyl group in the sphingoid base (Rodrigues et al. 2000). Eliminating this methylation alters membrane integrity and reduces virulence (Singh et al. 2012, Raj et al. 2017). Little is known about GlcCer catabolism, but a cryptococcal glucosylceramidase (EGCrP1) may act in GlcCer quality control (Ishibashi et al. 2012).

Within the plasma membrane of eukaryotes, distinct microdomains are enriched in ergosterol, GlcCer, other sphingolipids, and GPI-proteins. Such ‘lipid rafts’ are also found in *C. neoformans*, although they contain more saturated fatty acids (e.g. palmitic and stearic acid), fewer unsaturated fatty acids (e.g. oleic and linoleic acid), and none of the very long chain fatty acids (> 20 carbons) found in lipid rafts from mammalian cells and *S. cerevisiae* (Siafakas et al. 2006). Several virulence factors cluster in these domains, including the phospholipase Plb1 (Maruvada et al. 2012), the antioxidant Cu/ Zn superoxide dismutase (Siafakas et al. 2006), and the plasma membrane ATPase (Pma1) (Farnoud et al. 2014).

*Final thoughts* - The AIDS epidemic allowed the explosive emergence of opportunistic pathogens such as *C. neoformans*. The death toll caused by this fungus continues to be an enormous burden, especially in regions with limited health care resources. This impact, coupled with the challenges of drug cost, availability, toxic side effects, lengthy treatment regimens, and resistance, creates an urgent need for improved therapies.

*C. neoformans* is protected by concentric surface structures, each of which influences multiple aspects of pathogenesis. Unique features of these structures may offer targets for new antifungal agents, but many of them remain poorly defined. For capsule, we still do not know how GXM and GXMGal are arranged and associate with the cell, or most of the glycosyltransferases required for their synthesis. The mechanisms of capsule branching, shedding, and degradation also remain unexplored. Fungal cell wall synthesis is fairly well understood and has been successfully exploited for antifungal therapy by glucan synthase inhibitors, but this class of drugs is not effective against cryptococcal infection. Continued exploration of wall synthesis and regulation, and of cell wall proteins or strains defective in cell wall components that may act as vaccines, may help compensate for this gap in efficacy. Finally, the plasma membrane is the site of unique glycolipids whose synthesis and catabolism is yet to be fully elucidated; these and novel membrane proteins may offer targets for drug development or potential for diagnostics. Addressing these many fascinating questions, as we peel away the layers of this fascinating yeast, should lead to advances in fundamental biology and point the way to new ways to combat a formidable pathogen.
